# Creating conditions for effective knowledge brokering: a qualitative case study

**DOI:** 10.1186/s12913-022-08559-1

**Published:** 2022-10-29

**Authors:** Prue Burns, Graeme Currie, Ian McLoughlin, Tracy Robinson, Amrik Sohal, Helena Teede

**Affiliations:** 1 School of Management, RMIT University, Building 80, Swanson St, Melbourne, VIC 3000 Australia; 2 Warwick Business School, Scarman Rd, University of Warwick, Coventry, CV4 7AL UK; 3 Department of Management, Monash University, 900 Dandedong Rd., Caulfield East, Melbourne, VIC 3145 Australia; 4 Monash Centre for Health Research and Implementation, School of Public Health, Monash University, Level 1, 43-51 Kanooka Grove, Clayton, Melbourne, VIC 3168 Australia

**Keywords:** Process improvement, Knowledge brokering, Receptive context, Australia, Healthcare policy

## Abstract

**Background:**

Process improvement in healthcare is informed by knowledge from the private sector. Skilled individuals may aid the adoption of this knowledge by frontline care delivery workers through knowledge brokering. However, the effectiveness of those who broker knowledge is limited when the context they work within proves unreceptive to their efforts. We therefore need greater insight into the contextual conditions that support individuals to broker process improvement knowledge to the frontline of care delivery, and how policy makers and organizations might generate such conditions.

**Methods:**

Our research took place in a healthcare system within an Australian State. We undertook a qualitative, embedded single case study over the four year period of a process improvement intervention encompassing 57 semi-structured interviews (with knowledge brokers, policy makers, and executive sponsors), 12 focus groups, and 137 h of observation, which included the frontline implementation of actual process improvement initiatives, where knowledge brokering took place.

**Results:**

We identified four phases of the process improvement intervention that moved towards a more mature collaboration within which knowledge brokering by improvement advisors began to emerge as effective. In the first phase knowledge brokering was not established. In the second phase, whilst knowledge brokering had been initiated, the knowledge being brokered lacked legitimacy amongst frontline practitioners, resulting in resistance. Only in the fourth and final phase of the intervention did the collective experience of policy makers result in reflections on how they might engender a more receptive context for knowledge brokering.

**Conclusion:**

We highlight a number of suggested actions that policy makers might consider, if they wish to engender contextual conditions that support knowledge brokering. Policy makers might consider: ensuring they respect local context and experience, by pulling good ideas upward, rather than imposing foreign knowledge from on high; facilitating the lateral diffusion of knowledge by building cultural linkages between people and organizations; strengthening collaboration, not competition, so that trans-organisational flow of ideas might be encouraged; being friend, not foe, to healthcare organizations on their knowledge integration journey. In sum, we suggest that top-down approaches to facilitating the diffusion and adoption of new ideas ought to be reconsidered.

[Bringing knowledge brokers in is] like bringing consultants in. I've seen so many [who have] walked through this door, with minimal effect, because we haven't provided an environment for the thought process to succeed. And people think there is something wrong with the [knowledge broker]. Well, no, [they] are actually the experts, but what are we going to put in place? Again, it's what environment are we going to create for this to be successful? So that's the question you need to ask … Because if you don't create the environment for it to succeed, we'll fail and [move] on to the next thing. (Executive Sponsor of Knowledge Brokers, Participant 4).

## Background

Process improvement refers to the methodologies used to improve the quality, safety, and efficiency of healthcare service delivery by optimizing the means (i.e. processes) by which healthcare services are provided. Examples include lean management systems, Six Sigma, and Robust Process Improvement [1]. Because process improvement knowledge comes from the world of business and has origins of non-clinical descent, importing this knowledge into healthcare contexts can be challenging, and involves supporting ideas to traverse disciplinary and organizational boundaries [2–6]. Thus, while the value of process improvement knowledge is widely accepted and promoted by healthcare policy makers, its take-up in everyday practice is limited [1].

Knowledge brokering – an activity described as “get[ting] the right knowledge into the right hands at the right time” [7] – is often offered as a panacea to support the mobilization of non-clinical knowledge into health systems, including management knowledge such as that related to process improvement [2]. Yet, viewing knowledge brokering as “an unquestionable enabler of evidence-based practice” is unwise, because knowledge brokering is a complex and challenging pursuit, replete with “tensions” [8]. Genuinely embedding new knowledge into practice, and sustaining new practice, is difficult [8].

Broadly speaking, knowledge brokering involves importing knowledge into new contexts, with the view to facilitating the adoption and absorption of that knowledge. Knowledge brokers act as skilled conduits of knowledge, facilitating its transplantation across organizational, disciplinary, professional, sectoral, geographic, and cultural boundaries. Although there is no “consensus” on the actual role or activities a knowledge broker undertakes [9], and indeed these activities range broadly [10], a number of skills are generally agreed as crucial, and inventories exist. Kislov and his colleagues [8], for example, conducted an inventory of the skills identified in empirical studies as typical or desirable of knowledge brokers, arranging these into three categories: information management; linkage and exchange; and, capacity building. “Information management” includes skills such as project management skills, IT skills, and the ability to appraise evidence. The “linkage and exchange” category includes networking, negotiation, and mediation skills. “Capacity building” refers to skills such as teaching, mentoring, change management skills and the like. However, Kislov et al. [8] note that some of these skills and activities do not necessarily lead to desired outcomes, but can instead engender difficulties for knowledge brokers, such as a propensity for “doing” instead of “facilitating" improvement work, which is counterproductive to the aim of transferring knowledge.

In a vein similar to Kislov and his colleagues’ work, other studies emphasise the sociological nature of the skills needed to successfully broker knowledge. Burgess and Currie [7], for example, maintain that knowledge brokers must have the capacity to identify novel connections across bodies of knowledge and see new possibilities for application [7], such as the potential value of removing “waste” from patient flow processes. Translational skills, an important subset of the “expert facilitation” skills that are generally agreed to be vital [10], emphasize that knowledge brokers must use language, metaphors, and examples that help adopters make sense of new ideas and their potential applicability [9, 11]. Relatedly, effective brokers often possess diplomatic or political skills, since oftentimes knowledge brokering is a means of operationalizing organizational strategy, which can intrude into medical professionals’ jurisdictional domains. That is, knowledge brokers help managers to implement managerial ideas that are important and advantageous to the organization, but not necessarily to the profession of the intended adopter [7]. Brokers must also possess an understanding of how to socialize knowledge, to encourage its incorporation into practice and its institutionalization [11], which is why the importance of interrelationships, and networking and stakeholder management skills are often emphasized [9].

What is notable about the foregoing, prevailing understanding of knowledge brokering is that its focus is generally individualistic, and fixed on the behaviours and capabilities of knowledge brokers [2, 12–15]. The contextual conditions within which knowledge brokers and intended adopters are embedded, and how these conditions impact on knowledge brokers’ efforts, remain largely unexplored (although see Bate [16]). This is despite warnings that individual brokers are likely to have only a local and limited effect upon integrating knowledge into healthcare systems for process improvement. Moreover, it has been observed that even attending to trans-organisational arrangements, for example by establishing higher level agreements amongst like-minded bodies (e.g. Academic Health Science Centres) to mobilise and share new knowledge, does not necessarily result in that knowledge having an impact “on the ground” [17].

These developments suggest that something is missing in terms of our understanding of the everyday barriers that knowledge brokers face and how they might be supported in their work. It would seem important for those who wish to enable knowledge brokers, whether they be organizational leaders or policy makers, to attend to the wider organizational and contextual conditions within which process improvement is enacted, and to give greater thought to the impact that they might have on local context. The challenge, it seems, is to render these contextual layers more receptive to the efforts of individual knowledge brokers, so that the knowledge they broker might be institutionalised [5, 18, 19]. But this raises some important questions: What do we *mean* by “contextual conditions”? What do these conditions look and feel like? How do they manifest and how does one go about shaping them? Such knowhow would seem scant, in spite of the burgeoning research into knowledge brokering and its cognates, and in spite of the heterogeneous theoretical roots of these phenomena, which already include theories on contextual conditions and their effects on the diffusion of innovation.

The innovation diffusion literature is one of the most influential origins to which the concept of knowledge brokering may be traced [20, 21]; for an excellent and pithy review, see Green and colleagues [22]. Within this literature lies a stream of thinking that views innovation diffusion through an institutional lens (e.g. [23–25]). An institutional perspective on innovation diffusion takes as its starting point an understanding that individuals are embedded in a social context, and indeed are constructed by that context. Because human beings are so thoroughly socialized, it is difficult for people to think and behave in novel ways. Further, they are suspended in networks of interdependencies with like-minded people, all of whom are largely guided in their daily practices by shared informal and tacit factors, such as norms, values, and habits. Codified, tangible, or explicit rules, such as clinical guidelines, are less influential – on this matter see Gabbay and le May [26]. It is difficult to escape these informal factors, which essentially amount to behavioural pressures that favour the status quo. New ways of thinking and doing are to a substantial extent uncomfortable intrusions into the familiar, and possess no legitimacy [23, 24]. A consequential implication of this theorizing is that efforts to manipulate people’s behaviour (i.e. encouraging their adoption of a new idea, policy, technology, or guideline) by, for example, threatening to remove or promising to increase funding, is unlikely to generate genuine regard for, and engagement with, that which is new. In other words, cultural or social forms of control constitute a deeper undertow that is more powerful than managerial forms of control and regulatory apparatus [27]. That which is novel must gain legitimacy in order for it to be adopted in meaningful ways by meaningful numbers of people [28].

Precisely how new ideas might be legitimated through deliberate or strategic means is therefore a vexed issue. To a substantial extent, legitimacy contains a paradox, because legitimacy emerges once intended adopters gain comfort, familiarity, and trust with that which is being introduced [28]. This is why Edison, when designing the large-scale, systematized provision of electric lighting, mimicked as closely as possible the existing gas distribution system [24]. Intuitively, Edison realized that innovations must be “ground[ed] in … existing understanding” so that what is new nevertheless retains some familiarity with that which it is displacing [24]. The importance of attending to people’s sensibilities and aiding their sense-making in this manner is precisely why translational skills (e.g. careful attendance to language and metaphor) are vital to knowledge brokering.

While innovation diffusion can happen in unplanned ways [22], deliberately attempting to introduce and embed novelty into new settings – that is, attempting to legitimate novelty – is a process variously described by institutionally inclined theorists as a “struggle” [29], “fundamentally political”, [28], and “perilous” [24]. This is because innovators find themselves dealing with the “congealed tastes” [30] of social audiences [28] who ultimately determine whether an innovation is adopted. People’s “tastes” can harden in the face of naked, manipulatory efforts. People will, however, adapt their practices and ways of thinking when the context changes; indeed, they will contribute to the evolution of their own environments [31].

Context exists on a continuum and consists of both “distal” and “proximal” matter that is “never … settled” and ongoingly transformable [32]. Distal context can be discerned in matter such as far-reaching economic and social forces. Such forces might manifest as the mechanisms by which material (economic) goods are distributed, and the differential access to goods that these mechanisms produce. Distal context also includes the ideological discourses that shape a community’s values, and established institutions such as educational and professional institutions, which determine how labour is divided and the professional bodies and associations that are able to create and enforce rules for action (see Hall and Soskice, [33]). While these forces may be conceptualized as distal, they are sometimes said to be “working behind the scenes” at the proximal level; they affect every day empirical life [32]. They do so by manifesting in matter such as the frames people use to make sense of their everyday reality, the “arguments” and “negotiations” and “persuasions” they engage in [29], their habits, their “toolkits for action”, and their everyday interactions and practices [32, 34]. It is “on the ground”, to use Edelman and her colleagues’ phrase [17], where people confront the everyday reality of the institutional conditions in which they are embedded, where we see them grapple with their “local” or “proximal” context.

In sum, the literature on knowledge brokering has yet to delve as deeply as it might into the matter of context, and has generally sought to advance the field of knowledge brokering by focusing on the skills that individuals need for their immediate environments. The actual environment itself, and whether this might be malleable and amenable to intervention, has been given far less attention. An institutional perspective on innovation diffusion, such as we deploy, encourages a focus on constructs that help make both distal and proximal context visible and intelligible, and contains the conceptual tools needed to interrogate empirical data. In essence, contextual conditions impact on the effectiveness of knowledge brokering, which raises two important research questions we address: *What kind of conditions best enable knowledge brokering? How might such conditions be fostered by policy makers?* Through our empirical study detailed below, we educe a set of conditions that helped shape a policy intervention designed to introduce process improvement knowledge into a state-based healthcare system in Australia. Linking our findings to extant literature in the field of innovation diffusion studies, outlined above, we generate theoretically transferable lessons for policy makers concerned with supporting process improvement interventions across the globe.

## Methods

### Research design

We adopted a longitudinal, embedded, single case study design [35]. An “embedded” case study is one that contains multiple constituent parts with a focus on the context in which those parts operate [35]. As such, this design can accommodate three levels of analysis: the broader context, a “larger unit of analysis” and “sub-units” [35] or “subclasses” [36] of analysis. As foreshadowed above, the actual case or phenomenon that we studied is knowledge brokering, which occurred through a policy intervention designed to integrate process improvement knowledge into the public healthcare system of a state jurisdiction within Australia. The policy intervention comprised the larger unit of analysis within our study, while the implementation of the policy in various hospital sites comprised our embedded units of analysis. To align these units of analysis to our conceptualization of context, described earlier, we conceptualized context as existing on a continuum from distal to proximal. We conceptually positioned the wider context in which the policy intervention occurred (simply called “context” by many case methodologists) as the “distal context”. We positioned the policy intervention itself – our larger unit of empirical analysis – and the activities of policy makers as a mediating influence on context. Our sub-units of analysis (the actual sites where knowledge brokers attempted to implement process improvement initiatives) comprised the proximal or local context in which knowledge brokers were embedded (see Table 2).

An embedded single case study design is considered an appropriate, uncontroversial design in situations where understanding context is an aim of the research, or equally as important as understanding the detailed operation of the phenomenon of interest (in our case, knowledge brokering). The assumption underpinning this research design is that context interacts with and conditions practice, and therefore to understand this relationship the study of “sub-units” [35] or “subclasses” [36] of a phenomenon is just as important as the larger unit of analysis, as is the broader context. An embedded case study allows researchers to understand why [a particular intervention] has ended up as it has, where it is heading and what it may be able to achieve in the future [37]. Our research was conducted with the support of five industry partners, including four health services (comprising more than 10 public hospitals), who were our main source of sub-unit data for our study, and the government department responsible for the policy intervention at the centre of our study (our larger unit of analysis).

### “The Program”: The focal case

Established in 2008*,* The Program is an ongoing policy intervention that aims to improve the efficiency, effectiveness, and quality of care in hospital health services, by enabling hospitals to redesign care processes. This enablement work hinged on introducing process improvement knowledge into the jurisdiction, and encouraging its adoption by frontline medical professionals. At the inception of The Program, most health services possessed neither the capability nor the capacity to achieve the required improvements to care delivery; process improvement was novel knowledge to most frontline medical professionals. Recognising this to be a serious problem, policy makers sought to transfer new process improvement ideas and practices (specifically, Lean thinking and its derivatives) into 30-plus health services. Central to the policy intervention was the funding and training of full-time knowledge broker positions (henceforth, “Improvement Advisors / IAs”), who were embedded in participating health services. Policy makers commissioned the delivery of training in improvement methods and techniques for IAs, who were then tasked with building the improvement capacity of the clinical and managerial workforce in the health services where they were employed. IAs were supported by executive sponsors, who were drawn from the ranks of senior management and clinical leadership within the host health service. Critical to the policy intervention was its capacity building aims; it was not the intention of the policy intervention for IAs to actually do the process improvement work themselves, but rather to educate, guide, facilitate, and support frontline workers to improve the processes affecting their work. However, some IAs occasionally became “hands on” in their roles, and participated in or conducted some of the actual process redesign. Nevertheless, the ambition of the intervention was to achieve the wide-scale integration of improvement knowledge into frontline practice by enabling frontline medical professionals to lead improvement projects themselves. This was expected to generate sector-wide improvements and system-level impact, because manifold health services would possess the know-how to improve care delivery. However, when our research commenced in 2015, it was already clear via an independent evaluation that this expectation had not come to fruition [6]. This was due to a number of issues revolving mainly around governance, management, organizational culture issues, and the engagement of senior medical professionals. The lack of clinical engagement was reportedly an especial frustration of knowledge brokers. Some knowledge brokers connected clinical engagement problems to broader issues of organisational culture, citing insufficient expertise and understanding of process improvement at the governance and executive levels of the hospital in which they worked. At some hospitals, uncompelling leadership on the issue of improvement led to the intervention being saddled with a low-profile and no sense of urgency amongst staff, which stymied knowledge brokers’ efforts to engage their clinical colleagues. These matters proved significant barriers to embedding process improvement into clinical practice. While the individual projects within participating hospitals achieved meaningful returns-on-investment, the upshot of these challenges meant that the primary aim of the intervention, at this early stage of its existence, had not successfully transfused process improvement knowledge into the everyday practices of frontline medical staff.

To a substantial extent, participating health services were able to choose a “problem” for their designated knowledge broker to focus on, provided that that problem lent itself to solving through process improvement techniques. As a result, a range of local initiatives became part of the broader policy intervention. For example, some hospitals focused on improving their discharge processes, others focused on improving patient flow through the Emergency Department, or improving the patient experience and timeliness of outpatient services, and so on. While the actual improvement activity varied across hospital sites, in that hospitals chose to focus on differing process improvement projects, all sub-units of analysis offered the opportunity to analyse a particular kind of knowledge (process improvement knowledge) being brokered in practice. A crucial, further commonality binding the sub-units of analysis together is that they all operated within the same historical, institutional, cultural, and policy context – i.e., they all operated within the same broader context. These contextual factors affected the jurisdiction as a whole, not just one hospital within it. In methodological terms, the qualifying criteria for inclusion in the case were as follows: the hospitals brokered *process improvement* knowledge; and, they were part of the formal policy intervention, which meant that they all shared the same contextual conditions.

### Data collection

As detailed in Table 1, we collected several kinds of data at various stages of the intervention. We conducted a total of 57 semi-structured interviews with three groups of past and present intervention participants: policy makers; knowledge brokers, referred to throughout as Improvement Advisors (IAs); and Executive Sponsors (ESs). In larger hospitals, some IAs were well established in their roles and had undertaken extensive training prior to the intervention. They were seen as leaders amongst the IA community and absorbed the local responsibilities of the policy intervention on top of their existing improvement work. Many IAs, however, were relatively new to the intervention. Generally, they tended to be mid-career professionals, with either prior expertise in process improvement in non-health care sectors, such as finance or manufacturing, or a background in clinical fields other than medicine, for example nursing, speech pathology, occupational therapy, and so on. Many IAs had applied for a knowledge brokering role because they were looking to transition to a new career or a new sector. Policy makers generally had a background in some form of health policy, especially quality management. Some were career bureaucrats, others were new to policy making and new to process improvement; many possessed a clinical background other than medicine. Executive Sponsors were in wholly senior managerial roles within the hospitals participating in the intervention; nearly all possessed clinical backgrounds, and most were medical professionals, from a range of specializations.

**Table 1:** Methods and stages of data collection

2008 – 2014 (Intervention commences in 2008)	2015 (Research commences in 2015)			2016 – 2017 (Bulk of empirical work conducted)			2018 (Research concludes in 2018; Intervention is ongoing)			TOTALS		
Empirical research yet to commence	Analysis of contemporary and historical policy documents						Analysis of contemporary policy documents					
	Semi-structured interviews with past & present intervention participants			Semi-structured interviews with present intervention participants			Semi-structured interviews with present intervention participants			**Semi-structured interviews**		
	Policy-makers:	Improvement advisors:	Exec. sponsors:	Policy-makers:	Improvement advisors:	Exec. sponsors:	Policy-makers:	Improvement advisors:	Exec. sponsors:	**Policy-makers:**	**Improvement advisors:**	**Exec. sponsors:**
	4	13	6	10	10	5	2	7	2	**16**	**30**	**13**
										TOTAL: 57 interviews*		
	Focus groups: 8			Focus groups: 4						**TOTAL: 12 focus groups**		
	Field work: 9.5 notated hours			Field work: 99 notated hours			Field work: 28.5 notated hours			**TOTAL: 137 field work hours**		

^*^Three knowledge broker interviewees had also previously been involved in the intervention as policy-makers. We treated each of these cases as two distinct interviews, as we explored both sets of these participants’ experience. We also interviewed four key participants at multiple time points. In sum, we conducted 57 interviews with 49 individuals

Observational fieldwork also formed an important pool of empirical data, with fieldwork yielding 137 notated hours of data collection. We also conducted 12 focus groups with participants from across the policy and hospital system. These were typically integrated into industry events after the researchers had had an opportunity to present data and early findings, so we could ascertain the validity of our emerging interpretation. Some of the focus group participants had also participated in semi-structured interviews, and were from the groups of IAs, policy makers, and ESs described above. Many focus group participants had not participated in interviews, and were IAs from hospitals across the jurisdiction. The bulk of the fieldwork occurred after we had gained the trust of our participants, but the timing was also determined by events happening within the various improvement initiatives, especially if these revealed telling information about the engagement of intended adopters, and changes to everyday practices.

In terms of recruitment, for the semi-structured interviews recruitment initially took place via introductions from our government partner and our four health services partners. The email approaching participants was typically issued by a Chief Investigator of the project, after permission to do so was granted by a senior person within the hospital or government Department. Initial email contact informed potential participants of the purpose of the research, advised them that permission to approach the potential participant had been granted by a senior officer within the organisation, but that participation in the research was entirely voluntary. Recruitment of participants was not confined to our partners; we recruited widely across the jurisdiction, via a mix of introductions to other health services provided by our partners and a snowballing technique, considered appropriate when researchers seek participants who are able make “information-rich” contributions [38, 39]. We endeavoured to recruit medical professionals at the frontline, but this proved challenging, due to their professional priorities and limited availability away from clinical practice. We were, however, able to directly observe interactions between IAs and medical professionals, as IAs attempted to broker knowledge.

Data collection stages were determined in accordance with the aims of our research, and via a mix of pragmatism and empirical opportunity. As per Table 1, the research commenced in 2015 when funding and key research personnel became available. As well as conducting semi-structured interviews, focus groups, and observational field work, we also collected a wide range of policy documents and drew on industry literature from organisations such as the Health Foundation and the Institute for Healthcare Improvement (IHI). The purpose of doing so was to gain an historical understanding of the emergence of process improvement within healthcare, globally (at the distal level, we might say), so that we could then connect this, temporally, to relevant local events occurring within the jurisdiction we studied (see Table 2). Given the Program commenced prior to our study, we reconstructed its early history via interviews with key stakeholders involved in its initiation, and historical policy documents. We then re-entered the field at key stages of the intervention to capture shifting perceptions, activities, and effects. Field re-entry was guided by a mix of factors (empirical opportunities, for example), but the overarching concern was to meet the aim of the research, which meant eliciting insight into the conditions affecting knowledge brokering at the local or proximal level. This led us to seek out the three different streams or levels of activity displayed in Table 2: a) the distal contextual conditions generating an awareness of process improvement (and related) knowledge across the healthcare sector and the impetus to import and apply this knowledge within the study jurisdiction; b) policy-level activities to facilitate the importation and application of this knowledge; and c) frontline knowledge brokering experiences of the proximal context in which knowledge brokers were embedded. By way of example, state-wide policy intervention events (e.g. conferences and training events) and milestones (e.g. projected mid-way points of local initiatives across multiple hospital sites) provided opportunities to gather data concerning policy-level knowledge integration activities. For example, we attended reflective gatherings at various stages of the intervention as a whole, where knowledge brokers from multiple hospitals shared their learning with each other, presented to each other on the progress or outcomes of their initiatives, and received further training on process improvement. This afforded us insight into both policy-level activities and knowledge brokers’ experiences. At the sub-unit level, important hospital site events, such as initial meetings and workshops to roll out a new process, or team debriefing meetings on the progress of an initiative, provided opportunities to collect data on the everyday knowledge brokering experiences at the front line.

**Table 2:** Contextual conditions, policy-level knowledge integration activities, and frontline knowledge brokering experiences

	**Timeline**	**PHASE 1 (pre-2008)** **Drivers of change, the beginning of an innovation discourse, and an unprepared local context**	**PHASE 2 (2008 – 2011)** **Early implementation, a partial shift in policy approach, & the problem of legitimacy**	**PHASE 3 (2012 – 2015)** **Adolescent implementation & the problem of coordination**	**PHASE 4 (2016 – 2018)** **Maturing implementation & emergent collaboration**
DISTAL CONTEXT	**Contextual conditions**	• Burgeoning interest in QI methods for healthcare, globally; growing interest in organization theory. Business process re-engineering at Royal Leicester Infirmary gains renown. • Rising influence, domestically, of organization theorists & international boundary organizations specializing in improvement (e.g. Institute for Healthcare Improvement). • Domestically, public hospitals in deep financial trouble; rising urgency to curb expenditure & improve value. • Federal domestic performance targets established to improve patient flow through EDs & surgery; Australian hospitals experiment with Lean.	• Influence of organization theorists on healthcare performance improvement organizations grows. • High Performance Work Systems & Lean-inspired healthcare interventions flourish, internationally. • Domestic interest in improving value & efficiency heightens. • Domestic Lean networks and Lean boundary organizations capture policy interest. • Growing local discontent with poor research impact fuels domestic interest in research translation & knowledge integration. A knowledge translation & integration movement emerges.	• Healthcare is fastest growing area of domestic government expenditure; hospital expenditure is greatest within healthcare funding envelope. • Government concern with waste peaks. • Seed funding provided to establish domestic research translation centres and accelerate knowledge translation, particularly in hospitals. • Research translation centres facilitate greater collaboration between universities & health, but integration with government health departments and policy-makers is poor.	• Maturing conversations about knowledge translation, mobilisation, brokering, and integration. • Importance of research translation and knowledge integration gains recognition at Federal policy level. • Additional research translation centres established. • Push for greater transparency of hospital performance across a range of measures; push for innovation, rather than greater capital investment, to solve hospital capacity issues.
	**Policy-level ‘knowledge integration’ activities & events**	• Perceived need for hospitals to acquire improvement capabilities. • Perceived need for hospitals to address lack of critical thinking skills in frontline staff, and lack of mechanisms for staff to challenge taken-for-granted, non-value-adding processes. • Perceived need for greater efficiency and waste reduction, although no *“burning platform”* (ES P1) to reduce waste. • Distant rather than local searches for new knowledge carried out. Ideas parachuted in. • Early efforts to build basic process mapping, process redesign, and project management skills, facilitated by the funding of multiple, small improvement projects (*“products”*, not an holistic approach, PM P9). • Top-down approach to improving hospital performance through target setting and performance monitoring (the use of a *“stick”*, PM P9).	• Policy intervention commences (2008). • Policy strategy is to differentiate the intervention from already well-embedded, potentially synergistic programs and methodologies (e.g. quality improvement). • Rationale for intervention betrays a strong interest in improving efficiency. • Lack of enthusiasm amongst hospitals leads policy-makers to dangle funds untethered to outcomes, to encourage engagement. • Substantial involvement of Lean-inclined industry & consultants in shaping & governing of the intervention. • Improvement leader roles funded & embedded in hospitals; training in Lean techniques commences; performance improvement work carried out via multiple projects within participating hospitals. • Industry internships, site visits & mentoring for improvement leaders commence. • Improvement leader network established. • Extensive suites of Lean-inspired tools developed & shared across improvement advisor network. • Improvement capability framework for hospitals developed. • “*My original and to this day strong recommendation, which wasn't taken up, is that the quality managers and the people working in quality in the hospitals should have been the targeted personnel for this [process improvement] training and this capability uplift. Because, to my way of thinking, it's the same family of theories*.” (Policy-maker, Participant 43).	• Policy intervention evaluated (2012). Discrete project successes identified, but clinician engagement, organizational capability, fragmented knowledge integration & poor diffusion of ideas identified as issues. • Government restructure brings policy intervention together with clinical networks & leadership development activities. • Organizational improvement capability tool rolled out. Focus on organizational capability intensifies: “*It’s complex work, it’s not simple, because we’re trying to change the way organizations run, not just attack [performance] targets.”* (Policy-maker, Participant 2). •“*We saw ourselves as facilitating and coordinating and supporting the hospitals to build their capability to improve.”* (Policy-maker, Participant 9). • Training programs in process improvement for clinicians continue. • Review of public hospital capacity conducted (2015). Improvement capability (rather than capital investment) identified as key to sustainability of hospitals. • Improvement Advisor roles continue, but concerns emerge regarding impact and value of these roles. • Improvement clearinghouse is established.	• Further policy restructure takes place in light of recent reviews. Policy functions of improvement, safety & hospital capacity brought together, as policy integration and synergies are sought. • Intervention survives, with new emphasis placed on: • Clarification of roles and expectations of Improvement Advisor positions. • Appointment of specialized “industry coaches” (specialists in process improvement) to work alongside Improvement Advisors, but with new conversations about knowledge brokering issues. • Engagement with Improvement Advisors to develop individual capability-building framework that extends beyond building technical knowledge and mastery of technical tools. • Engagement with Improvement Advisors to re-develop organizational improvement capability framework for hospitals, so that improvement knowledge can be better exploited. • Facilitation of peer-to-peer mentoring amongst improvement advisors through strengths-appreciation process that teams up experienced & inexperienced Improvement Advisors. • Establishment of an Improvement Advisor community of practice, resulting in open sharing, including of failure: *“Initially, you could see people walked in, they stayed in their groups …. You know, we had to work really hard to facilitate it and it tended to be quite didactic. Now, it is completely different … And although we may have shifted some individuals, there are still some challenges for some health services … because actually, in sharing, you're sharing your failures as well as your success, otherwise it's not sharing. And a number of health services had real problems with that concept of sharing failure.”* (Policy maker, Participant 34). • Cross-hospital knowledge-sharing via centrally-coordinated, cross-hospital networking & system-wide showcase events. • Use of social media to communicate & enhance profile & discoverability of local improvement learning. • New emphasis placed on looking locally for improvement inspiration and mobilizing local know-how.
PROXIMAL / LOCAL CONTEXT	**Frontline knowledge brokering experiences**	• Hospitals exposed to basic project management skills and rudimentary process improvement tools. • Concept of knowledge brokering and knowledge integration unestablished within jurisdiction. • No sense of urgency for reform amongst frontline staff, leading to desultory process improvement attempts targeting only *“low hanging fruit”* (Executive Sponsor, Participant 1). • Basic skills required to prepare workforce for required improvement: *“The main goal [from my perspective] was to increase capability and capacity to be able to respond to problems that [front line staff] identified. But the challenge was you didn’t have a workforce that even asked questions and solved problems.”* (Executive Sponsor, Participant 1).	• Project approach becomes wearing on frontline staff, with Improvement Advisors bearing the brunt: *“Death by a thousand projects” seems to be a familiar refrain among Improvement Advisors.* (Field note). • Improvement Advisor network established, and successful in terms of circulating knowledge throughout this network. At the same time, collaboration between health services is seen as unusual: *“That network means that there’s a culture in [improvement] of sharing. That’s unusual [here] in health – it’s crazy, but it’s unusual.”* (Executive Sponsor, Participant 14). • *“I think broadly from [policy-makers, the ICPH in its early days] was [about] seeing health organizations tooling up.”* (Improvement Advisor, Participant 7).	• *“My experience of watching [hospitals] go Lean is that after a point in time your staff do a backflip and start to resent it:* ‘Here come the Lean people’*”.* (Improvement Advisor, Participant 4). • *The Department should actually be building their policy knowledge based on the [local] issues that appear in health systems. And they don’t necessarily to the extent they could. It’s a power shift. So, do with, not to. So that’s the shift that I would see should be made.”* (Improvement Advisor, Participant 13). • *“I think [the IA network has] run its race in [terms of] being a supportive group, for a group who are thinking about,* ‘Maybe I’ll do this [improvement] thing’*”.* (Improvement Advisor, Participant 7). • Improvement Advisors report difficulties in engaging clinicians in process improvement and encountering receptiveness issues. • Competitive nature of system openly acknowledged by Improvement Advisors, Executive Sponsors, and policy-makers.	• *“By the time we got to the third [collaboration event] it opened right up because people started talking about their problems. We started to realize that actually, the issue you've got here at [this health service] is the same issue as [over there]. And [that other health service] has just recently solved that same issue as well, and we start to see this more collegiate kind of thing happen. For the most part, I think they've got these relationships now where everyone will pick up the phone and talk to each other.”* (Improvement Advisor, Participant 48). • Improvement Advisors begin to express mixed opinions about the competitive nature of the system, and can instead point to examples of collaboration that extend beyond the Improvement Advisor cohort.

### Data analysis

While our research design was informed by theory on the institutional context and the role this plays in enabling or constraining innovation practices, we began immersing ourselves in the more specialised literatures of knowledge brokering and innovation diffusion after the design phase of our research. We realized through our post-interview team debriefs that these latter literatures would give us theoretical purchase on our empirical data, and would likely be instrumental in later efforts to theorise our findings. We therefore began our analysis of the data with a theoretically-informed coding procedure to elicit insights at two levels of analysis: the activities and efforts of policy makers to mediate the local context and support knowledge brokers; and, the actual brokerage of improvement knowledge into frontline practice. By this we mean we did not endeavour to set aside the a priori theoretical constructs we had earlier acquired through our reading, such as notions of legitimacy, adopters’ responses to novelty, social boundaries, credibility, the “in-between” position of brokers [8], and so on. Instead, we fragmented our data into initially descriptive codes, and then later collapsed these into fewer, more abstract codes informed by the theory, in a process partially reflective of “template analysis” [40]. By way of example, “responses of social audiences (medical professionals) – ignore broker”, was a code emerging from our field notes. We did not force our data into pre-existing theoretical constructs, however, and we did not discard it if at face value it did not appear to fit comfortably into existing theoretical notions as we wished to avoid treating existing theory as “received wisdom” [41]. Instead, we collapsed similar codes into more distilled, secondary codes expressed in lay terms, for example, “respecting local experience”. This coding procedure provided us with a platform for both respecting the existing literature and extending it with new, empirically-informed constructs. Thus, our coding process was simultaneously a search for both novelty and fit between our data and existing theory, with the view to challenging, if necessary, the existing theory.

As can be seen above, during our initial coding process we dropped any regard for temporality. However, to help us identify the interaction between context and knowledge brokering on the frontline, and to be able to attribute causality between context and frontline practice (i.e. contextual conditions “A, B, C” lead to frontline approach “X”), we needed to be able to see relationships along two dimensions: “horizontally”, meaning relationships between sequences of events across time; and, “vertically”, meaning relationships between distal and proximal layers of context. Thus, we established a matrix to aid our second stage of analysis, and drew on a combination of two of Langley’s [42] techniques for analysing process data, both of which are conducive to unearthing patterns and mechanisms driving change, and therefore theorizing relationships between context and outcome patterns [41]: Langley’s visual mapping strategy, and her temporal bracketing strategy.

To begin, we drew on Langley’s “temporal bracketing technique” to identify concrete objective events and used these as points for allocating timeframes [42]. This resulted in four phases being established: *pre-intervention (pre-2008)*; *commencement of early intervention and early years (2008–2011)*; *post-evaluation of intervention (2012–2015)*; and, *maturation of intervention (2016–2018).* We then streamed our insights from industry and policy documents, plus our coded constructs into these epochs to allow evolutionary phases of the intervention to be plotted against a time line. Simultaneously, we channeled our coding into three broad levels of analysis: 1) a distal contextual level, to make sense of the higher order environmental conditions affecting the work of policy makers and IAs; 2) a policy level, to identify and chart over time concrete knowledge integration policy activities; and 3) the local or proximal contextual level, which contained codes pertaining to the lived experiences of those actively involved in knowledge brokering. We mapped these layers across our temporal brackets, which allowed us to match people’s lived experiences (or their reflections of their lived experiences) of knowledge brokering to a given time-frame and specific context. A vast matrix resulted, which allowed us to see patterns in our data, and trace potential causal connections between higher order contextual conditions, policy activities, people’s experiences of knowledge brokering, and the perceived effectiveness of their brokering efforts. As a final analytic step, we concentrated the data within the cells of our matrix into slightly more abstract, summary observations and allocated more formal labels to these phases (see Table 2). The resulting vantage point allowed us to derive bounded theoretical (as distinct from statistical) generalisations via a small number of “thought experiments” [43]. We conceptually positioned distal context as an independent variable with flow-on effects to policy-level activity, and we conceptually positioned policy makers as a mediating variable with flow-on effects to the proximal context where frontline knowledge brokering took place. We then created a number of abstracted claims or principles based on our codes as to how policy makers might mediate context. For example, the observation from our coding that knowledge brokers and adopters appreciate their extant, local knowledge being respected, was transformed into *Pull knowledge upward; do not solely push knowledge downward.* Guided by George and Bennett [36], we conceptually tested our claims by asking ourselves questions such as, *Do any of our empirical data contradict our claims? Might better rival explanations exist? Is it plausible that these mediating policy-activities will render the local context more receptive to knowledge brokering, based on our data pool as a whole?* When we identified instances, events, or experiences in our data that contradicted our claims, we dropped or reformulated our claims and then “re-tested”, conceptually, whether the claim held. An important part of our efforts to check the credibility of our conjectures was a number of “member-checking” fora [44], where we reported on our emergent thinking to our participants. In sum, our analytic procedure might be described as a process of working increasingly toward higher levels of abstraction or generalisation, with criteria such as accuracy (in terms of reflecting the data), plausibility, and generality guiding the formulation of our statements.

## Results

Table 2 sets out a summary of the phases educed from the data, with supplementary illustrative quotes to show how policy makers sought to shape the local level context, and how IAs sought to enact their knowledge brokering role. We provide a narrative of these phases below.

### Phase 1: Drivers of change, the beginning of an innovation discourse, and an unprepared local context (Pre-2008)

In the early 2000s, policy makers responsible for the performance of the state’s public hospitals faced a daunting task: find new ways to make healthcare service delivery more efficient, but keep improving the quality and safety of care. A confluence of factors had sparked this imperative: emerging financial pressures; the imposition of national hospital performance targets; and the enormity of the impending demands of an ageing population. In light of the coming strain, policy makers started to critically scrutinize existing models of hospital care delivery, and the seed of a transformation agenda was sewn: build the capability of frontline healthcare professionals to fundamentally redesign business-as-usual mindsets and models.

Around this same time, healthcare policy makers in Europe, the US, and neighbouring New Zealand were already in the process of experimenting with business ideas, and applying these practices and techniques to hospital settings [45]. The promise of these ideas lay in their demonstrated ability – in other sectors – to create value, deliver efficiency, and improve quality for consumers. When stories emerged from the UK of the successful application of business process reengineering to *hospital* processes [45], pioneering policy makers in Australia began agitating for, and secured, funds to build basic project-management capability at the frontline, in preparation for trialing some of the more rudimentary process improvement tools in use overseas:

We had $15 million and we had about 83 projects and [our program] was based on work at Leicester Royal Infirmary in the UK. So, we had a project manager who’d come out from the UK and set this program up, and basically it was very basic process redesign. It was about building capacity in the health sector for very basic process redesign, and we taught facilitation skills, how to do process mapping, how to build a project team, how to run a project properly, what sort of people you need in it, those sorts of things. (Former policy maker, Participant 9).

These initial efforts were described by policy makers who were involved at the time as piecemeal, *“product focused”* (Former policy maker, Participant 9), and a top-down, target driven approach to building capacity and diffusing new ideas. It was acknowledged that this approach touched relatively few frontline professionals, and it was described in subsequent policy evaluations as *“fragmented”* and *“short-term in approach”*. The intent of policy makers to build the improvement capacity of frontline staff was genuine, but there was little understanding about how to reach deep into hospitals where everyday, taken-for-granted practices took place, and how to establish the local foundations to support hospitals to change these practices:

So everyone goes straight to the outcomes of a high performing health service. I say, “No, no, what are the foundations? What are the things that sit underneath that make this service high performing?” The results are just the outcome of that. The emergency targets, the elective [surgery] targets, they’re just the outcomes [you want], but you need to have leadership, you need to use data well, you need to have an improvement methodology, you need to… these sorts of things … There’s a lot of people in the Department who aren’t very strategic, even though they’re looking at the whole health system. They look at products, let’s create this next product. And so what I used to do was create products for them. (Former policy maker, Participant 9).

Activity at the policy level was building, focused on how to generate and diffuse innovation within the hospital system. Ministerial councils on hospital innovation and other working groups were established, and executive leadership development programs for senior hospital staff were established. Within political and policy circles, a process improvement discourse had begun.

However, the arenas this discourse penetrated were limited, and they did not form part of everyday conversation amongst intended frontline adopters. Instead, discussion about redesign and process improvement sometimes took place within the higher management echelons of larger hospitals (in part due to the leadership programs that had been established), but only very occasionally did conversations about process improvement reach into clinical circles. When they did, this was reportedly well-received by clinicians:

Apparently general engagement between health services, policy makers, and clinicians improved markedly around the turn of the century when basic improvement capability building was taking place. This engagement seemed to be appreciated by at least some clinicians: *“Partnerships”, “building conversations”, “establishing trust”, “making acts of commitment”* – the senior clinician at the table identifies these things as crucial to the process improvement work that had previously saved their health service $12 million. (Field notes from a think tank meeting with health service senior executives, senior clinicians, ESs and IAs).

Clinician engagement, however, was exceptional – it was something that the hospital executives and senior clinician in the fieldnote, above, were proud of precisely because it was unusual. In general, it seemed there was an assumption amongst policy makers that the ideas and techniques of process improvement could be mobilized and diffused via top-down waves of policy and organizational rules, enticement mechanisms such as increased funding, and the wielding of a *“stick”* (Former policy maker, Participant 9) in the form of performance targets:

Hospital performance became really big and very targeted and you had targets and you got measured and you got scored and you got put on performance watch if you weren’t performing and all those sorts of things. (Former policy maker, Participant 9).

The prevailing policy viewpoint was highly functionalist in nature, and assumed a centralized, command and control style of policy implementation would successfully filter through hospital management hierarchies and that people working at the frontline would see the value to their patients in re-imagining hospital processes. It saw hospitals as being *“driven from the top”* (Policy maker, Participant 24). Within this paradigm, parachuting in people, ideas, and expertise from overseas made good sense. What it failed to fully appreciate, however, was the context where the policy rubber hit the road. The everyday pressures of providing clinical care, the power of clinicians’ professional identity and their jurisdictional boundaries, the freedom from oversight they enjoyed, their valued autonomy and lack of connection and commitment to hospitals as formal organisations – these things were not front and centre of policy makers’ minds. For medical professionals, the idea of directing energy toward improving hospital *processes* was largely foreign and had no pragmatic value to them. Understanding how to improve workplace processes was not part of their professional training, responsibility, or interests. As one ES explained, when asked about the challenges of engaging medical professionals in process improvement, *“clinicians have a day job”* (ES, Participant 4). This was a matter-of-fact observation. The ES went on to explain that, *“a lot of them just come to work, do their thing, do a very good job, look after their patients well”.* However, professional associations and colleges – the major socializing force affecting clinicians and determining the kinds of knowledge that matter to clinicians – paid little heed to matters concerning hospital processes, hospital governance, and financial pressures:

I think that's one of the biggest challenges in health, compared to other industries. When it comes to the reality of the scenario you're in, in a healthcare system very little of [what’s needed] you're taught at the undergrad level. Health policy, health economics. You don't get taught any of that stuff as a clinician. So [redesign and the like] is actually not natural to you … And the training [you get as a clinician] is very much one-patient-at-a-time, which is important stuff but, they don't get taught all the [other] stuff that really matters. So, yeah that's a space that as a health system we need to plan. And [medical professionals] don't get taught governance. So the health system needs to play a very strong role in terms of education in that space. (ES, Participant 4).

In general, then, policy makers appeared to have little reach into the spaces where professional development and practice took place. There was relatively little awareness or interest amongst the intended adopters of improvement, and reportedly no felt sense of urgency to direct their attention to organizational processes. As one ES explained:

Now unless you have an absolute burning platform and a total transformation or agenda it’s hard to galvanise the whole organization … In New Zealand, finances was the burning platform because they are much smaller than Australia and they don’t have minerals. So we were doing this stuff a lot earlier on around finances and efficiency, and a lot earlier than, say, Australia was. (ES, Participant 1).

Effectively, some participants believed there was insufficient internal pressure within hospitals to generate sufficient doubt about existing processes to change them. Frontline clinicians’ belief in existing ingrained frontline practices had no experiential reason to be questioned. The top-down imposition of pressure, through performance targets, was seen as something for executives to deal with; the new policy discourse regarding improvement had little meaning at the local level, where practices needed to be changed.

### Phase 2: Early implementation, a partial shift in policy approach, and the problem of legitimacy (2008–2011)

Leveraging the learning and resources that came before it, the Program formally commenced in 2008, with funding secured to employ, train, and embed into 16 of the earliest participating health services the first two waves of IAs. The Program was informed by the precursor initiatives described earlier, but it was pitched as a new solution and carefully distinguished from prior innovation efforts. The centrepiece of the Program was marketed as the injection of a new kind of role, new capabilities, and what was described as *“new ways of thinking”* (IA, participant 52) into health services. Distinguishing process improvement from earlier efforts seemed to emerge from a desire to jettison any preconceived, negative ideas that people might have about the Program and to play up its novelty, which it was assumed would generate interest:

I think we’re indoctrinated into the only way you can engage is to make it sexy and look nice and it’s new, it’s a new thing. Because it’s really boring to go back and say, “Oh we’re just going to keep working on the basics”. (Former policy maker, Participant 9).

Playing up the newness of the offering, however, had important ramifications, and ultimately was unsuccessful as an engagement ploy. It painted the intervention as something strange and unfamiliar, and it differentiated the Program from more established ways of improving clinical practice, such as quality improvement. It was assumed that differentiating the Program would be advantageous, but this assumption proved untrue. The Program was more difficult, not less, for clinicians to relate to (whereas they were familiar with the prior program and quality improvement activities), complementary skill sets at the policy and hospital levels were not leveraged, and potential allies (e.g. quality assurance specialists) were antagonized, who were otherwise well-placed to help introduce new roles and skills into health services:

There was a real tension when this first came [in] between what was [process] redesign and the bods that are traditionally in that quality space. (IA, Participant 19).

In all, niching the Program validated a pre-existing belief amongst frontline staff that process improvement (and similar activities) fell outside their professional domain – in a sense, this was the opposite of what was needed to engage frontline staff. The Program’s core aim was to build the capacity of frontline clinicians to actually lead and conduct process improvement. Yet, similar to its precursor forms, the focus of the Program was on gaining abstract, system-level efficiencies, which were far removed from the “thinking as usual” of medical professionals. As one IA explained, who was also a qualified medical professional:

I think sometimes because you’re used to a particular way of thinking and I think a lot of doctors are, how should I say this? They’re used to their way of thinking and a lot of doctors are also quite intelligent in that they tend to think sometimes the way that they’re used to working in medicine is the right way, but sometimes actually there’s other ways of working, that you’re not necessarily always right or there’s other opinions to take on board. I think because of the way we are taught in medicine that when you see a disease, as such, that’s a problem that needs to be fixed or treated. So you tend to jump straight to, “How do I fix this?”, but in redesign it’s very much the opposite, which is you need to really uncover things and understand what are you trying to solve, here, rather than be reactionary to what you’re faced with. (IA, participant 52).

Similar to its earlier incarnation, frontline clinical staff also correctly perceived the Program as being animated by a strong *“efficiency remit”* (IA, Participant 7), imposed from on high. In many hospital sites, the Program was reportedly seen by frontline clinicians as embodying bureaucratic concerns about unmet hospital performance targets, which were divorced from their everyday concerns. In general, IAs and ESs reported that process improvement was perceived as a form of knowledge of little relevance to the skillsets of care provision, and at odds with their core values. Even some IAs and ESs, who were meant to be ambassadors for the Program, were skeptical of the applicability of process improvement methods to hospital processes; grave concerns were held regarding the lack of buy-in by frontline staff. Some ESs and IAs regarded the business provenance of process improvement as a nearly insurmountable hurdle:

There's a fairly big difference between a patient flow versus a car production flow. (Executive Sponsor, Participant 39).

I think the frustration with working with real life people rather than cars has been a challenge, and we can see how a standard would run beautifully in a place like a car factory or car manufacturing… Things have to just keep moving, or else the machine stops and that's basically what happens here if they don't move people. Our machine stops and we get a back up of cars out in the waiting room. (Improvement Advisor, Participant 23).

Policy makers were also aware of these difficulties:

Doing some of these changes, particularly when they’re coming from outside the health industry, so aviation or service industries like banking, that’s quite a challenge. People are, like, “We’re special and different, we work with people not widgets or cars.” (Policy maker, Participant 2).

The reports of IAs generally suggested that responses such as those noted above sometimes contained an implicit assertion that the work and knowledge of frontline clinicians is not only different from that within the world of business, but superior:

L]ike surgeons, or whether it’s individuals who have been in health … for a long time and so they have their way of working and they know what’s best … And they say, “Look, I actually don’t need your input, I already know what I’m doing. I already know how to do it, so thank you very much, off you go”. (Improvement Advisor, Participant 8).

Ironically, the policy approach to position the Program as something *“shiny [and] new”* (Policy maker, Participant 33) and the technical nature of the training provided to IAs (project management, IT skills, process improvement tools) had honed the *de-*legitimating features of process improvement. IAs’ main sources of advice and expertise came from outside of healthcare contexts, via participation in industry internships, visits to manufacturing sites, and mentoring from consultants from business, finance, and manufacturing industries. The aim of the Program was to embed process improvement into frontline clinical practice, yet IAs possessed no authority at the ground level, informal or otherwise, and the ideas they were charged with purveying had no discernible appeal to their target audience, Frontline clinical staff found work approaches that likened hospitals to factory lines and people to widgets distasteful and irrelevant. These issues reinforced rather than dissolved knowledge boundaries, and marginalized Improvement Advisors. The distance in understanding and status between IAs and clinical frontline staff was replicated in physical and symbolical ways, not just skirmishes at the clinical frontline. IAs were rarely embedded within clinical teams, and their offices were often located in the dingiest hospital buildings, far removed from clinical action.

Policy makers had actually anticipated and sought to counteract some of the difficulties described above. The funding of IA roles was not contingent on predetermined performance outcomes, and hospitals were granted considerable *“autonomy”* (policy document) to choose how they deployed their IAs. Essentially, taking place at the policy level was,

“… an interesting debate about whether we’re prepared to tolerate variation [in improvement projects and approaches across hospital sites] and whether we’re going to use a more centralist approach to drive them harder rather than it being self-taught, self-learning, learn from your mistakes, live through the agony and eventually you get there … I think that is interesting, there is an interesting balance and an interesting debate there about if we come and intervene, then you get resistance and will services want that? Or should we allow them to fail a few times until they learn themselves? (Policy maker, Participant 24).

Policy makers also tried to temper their earlier top-down engagement approach and shift the way they engaged with hospitals:

There was this discussion about what’s [our] role in [hospital performance]? Is the role to just monitor and hit you with a stick and tell you, “Here’s your targets”, and try and get performance that way, or is the role to fund projects and improvements and strategies and all of those sorts of things at the same time to help health services perform. So, we very much from the [new Program perspective] thought we saw ourselves as facilitating and coordinating and supporting the health services to build capability to improve, to do improvement, basically, and that was one of the fundamental things that we saw as our role. (Former policy maker, Participant 9).

And so in conceptualising the Program, it was really, go in at a grass roots level, try and train enough people to reach a tipping point that people would be interested and could see that there was an opportunity for improvement. (Policy maker, Participant 24).

Contained in these debates is an awareness that policy level activity has tangible effects at the local level. However, the success of these shifts was mixed. Some Program outcomes were certainly achieved, and earned the extension of the Program to more than 30 health services, and the training of further waves of Improvement Advisors. But the lived experience of IAs suggested that, at the local level, these successes were very hard won. Frequently, IAs reported encountering *“resentment”* (IA, Participant 4), *“aggression”* (IA, Participant 15), and *“defensiveness”* (IAs, Participants 4 & 16) at the frontline. So peripheral was the role of IAs, they identified being *“invited in”* (IA, Participant 26) to wards and units by clinical managers as crucial to the success of their improvement efforts. A threshold or boundary lay between IAs and the intended adopters of process improvement, with the latter working to keep knowledge boundaries in place, by actively distancing their work, ideals, and values from the knowledge that IAs were trying to broker.

### Phase 3: Adolescent implementation and the problem of coordination (2012–2015)

An evaluation of the Program in 2012 called for its continuation, but voiced concerns that it had produced few joined-up, systemic outcomes, and that the integration of process improvement knowledge was immensely variable across the hospital system. Whereas the early years of the intervention had focused almost exclusively on building the technical skills of IAs, the middle years of the intervention saw an attempt to rebalance this focus. Effort was invested in building knowledge integration and exploitation capabilities at the organizational level, and in fostering a familiarity and affinity for process improvement among frontline healthcare professionals, via an increased focus on training programs in improvement for clinicians, and the roll-out of a tool to help health organizations assess and build their knowledge exploitation and process improvement capabilities.

A subsequent hospital capacity review in 2015 reinforced this diagnosis, noting that the intervention had facilitated *“important but small projects”,* but that there was *“no system-wide gain”.* The perceived need for centralised coordination to join up improvement efforts across the system, and to do more with less, was a strong undercurrent throughout the report. Healthcare was the fastest growing area of government expenditure at the time, with hospital expenditure the greatest contributor. The need to build improvement capability was identified as key to the sustainability of the healthcare system. The intervention thereby secured a reprieve, and signaled a more active coordination role for policy makers in the future.

If not quite the *“burning platform”* fabled to spark frontline engagement, it certainly seemed a smouldering one. Yet at the local level, the financial exigencies that occupied the everyday reality of policy makers and hospital executives did not flow through to frontline clinicians.

By way of example, senior executives of one of the worst performing hospitals in the state, which had been excoriated and shamed on the front pages of newspapers around the country for spectacularly failing to meet Emergency Department (ED) service targets, had been trying for months to redesign its ED processes and accelerate patient flow. They had hired external experts to lend technical support and authority to their in-house Improvement Advisor. The IA had set up a Kanban board in the ED, to monitor the efficiency of the new processes that had been established. Yet:

People became totally biased against the whiteboard before it even started. Like people playing, you know, it was quite … I shouldn't say … But you know we had people playing overnight, they were playing battleship on it and noughts and crosses. And I'm sure, you know … And it wasn't being disrespectful, but it was just because we weren't really using it properly because no one knew how to use it. So it was a big white board. And what are you going to do? You're going to play noughts and crosses with it. (IA, Participant 32).

Despite more than a decade of sector-wide effort to generate respect for process improvement methodologies, it seemed that some intended adopters still judged these methodologies unimportant. In the same ED, during an audit of the new process that was being trialed, the Kanban board was largely ignored, and business as usual ensued:

Curiously, all this activity [in the ED] does not add up to a sense of urgency. Instead, it creates a steady thrum of ambient noise and constant, but generally unhurried, comings- and-goings. Leaving aside the way doctors respond to the Bat Calls (calls that alert the ED to incoming ambulances with patients in critical condition with a piercing triple beep), which is undoubtedly prompt but not frantic, it doesn’t feel in any way hectic in the bowels of the ED. Doctors scrutinise computer screens, enter details, discuss with each other x-ray and ultrasound results, patient conditions, difficulties getting wards to accept patients for admission, and lean back in their chairs or perch or sit on the edge of the benchtop desks and occasionally punctuate clinical conversations by regaling each other with amusing or annoying incidents as they occur. The energy and activity of the nurses is not too dissimilar from that of the doctors. On one occasion I hear a doctor and a nurse talking about bikes with training wheels; I time the tail end of the conversation; it’s just longer than 8 min. … Later, the process improvement expert, a consultant from the car manufacturing industry, who has been engaged to help the IA, asks me if anything has surprised me about the ED. *“Yes”,* I reply, *“It’s really relaxed. I thought it would be frantic.”* He laughs: *“Same! It’s not the picture I expected after being told how dire the situation is.”* (Field notes from a process improvement audit in the Emergency Department and subsequent meeting with consultant and IA).

While pressures to innovate may not have been felt by frontline clinicians, senior hospital executives generally felt them keenly, and were drawn in to the Program as far more active participants than in the past, with greater responsibility for creating organizational conditions that enabled process improvement to become part of business as usual. However, as one former policy maker pointed out, this remained a top-down approach to diffusion and embedment. While some effort had been made to draw medical professionals into the Program through specialised training programs, these efforts, too, were created and managed from policy, rather than professional, centres of authority. In sum, some participants suggested that the adoption of process improvement at the frontline, and peer-to-peer diffusion of process improvement did not occur by applying downward pressure. Ironically, collaboration and therefore diffusion seemed to occur via a lateral flow of ideas, across peer and professional networks:

[There were] waves of rollout – top down through the hierarchy and powerful actors, but this is not how collaboration amongst the frontline occurs … [T]he lower you go down, the better the interaction and the networking is, so [lower level staff], they’ll talk to each other and know who’s who in the zoo. But if they need permission of their executive to do it, they might not do it quite as much. (Former policy maker, Participant 9).

Problems with what participants called “collaboration”, which is key to the diffusion and embedment of new ideas and practices, were reasonably widespread. Participants were disgruntled about the competitive nature of the sector (*“There’s a whole lot of competition”* [Executive Sponsor, Participant 6]), leading to calls for stronger policy intervention to help distribute knowledge across health service boundaries:

“It’s such a pity that [policy makers] don’t take a leadership role in this sharing across health services. They just don’t.” (Executive Sponsor, Participant 14).

An IA network had earlier been established, but on the face of the two reviews appeared to have yielded little in terms of diffusing improvement successes, probably because IAs were essentially policy creations and wielded little influence within their health services. Without internal purchase, strong relational linkages between IAs and meritorious improvement ideas mattered little in terms of knowledge integration. Even those IAs involved in the network in its early, strongest days, conceded it was ineffectual and indicated a need for more directive intervention from policy makers.

### Phase 4: Maturing implementation and emergent collaboration (2016 – 2018)

The 2015 capacity review heralded a policy restructure, and a critical period for policy makers to review and secure the survival of the Program. Substantial policy changes and a determination to *“rejuvenate”* (Policy maker, Participant 37) the initiative ensued. The Program was enfolded into the quality and safety policy-making branch, to capitalise on the legitimacy of quality and safety, while maintaining the initiative’s unique value proposition. Crucially, policy makers reconsidered their own roles as stewards of the Program, and set about establishing more collegial, partnering relationships with IAs, in an attempt to dissolve the more formal boundaries existing between themselves and IAs. Instead of taking on purely program management roles, a partnering approach was adopted:

A challenge … is knowing how we work, and try and look at those partnering relationships as opportunities. Also, don’t lose our sense of what our purpose is and how we can add value. (Policy maker, Participant 33).

We're starting to get a reputation in being the helper. Linking people together. Leveraging the resource and the expertise that we have in our department. (Policy maker, Participant 36).

With Peta, Peta has developed such a beautiful relationship with [her Improvement Advisors] that they’re actually on the phone to her on weekly basis. She’s now having to withdraw a little bit, and let them fly on their own. (Policy maker, Participant 33).

It seemed that policy makers were reconsidering the way that ideas might be circulated; there was an understanding that mandating the sharing of ideas ran counter to the way that people self-organised, learned, and adapted:

So one of the things is health services are very large organisations and they’re driven by the top, but they’re also driven by the bottom. And so the way we train our workforce, the way consumers interact with health services, so you’ve kind of got to remember what consumers are demanding from health services also changes the way health services might perform their function. And there is a kind of clinical practice exchange, movement of staff across organisations. So there’s a whole bunch of ways in which information gets shared. It’s not necessarily by a report on an initiative being shared across five health services. Actually, it could be the movement of 500 nurses across Victoria every year because they just move house or wherever they want to move to. So the interplay between services I think, in terms of workforce on the ground, is also a mechanism that we shouldn’t forget. And that’s both informal and formal, so colleges, clinical networks, clinical guidelines, a whole bunch of things. But diffusion of [innovation] is not just at the top and it happens both formally and informally. And that can be good diffusion and bad diffusion as well because you can also have bad ideas shared across the system as well. (Policy maker, Participant 24).

Policy makers also became more circumspect about allowing themselves to be seduced or *“distracted by shiny new stuff* – *‘Oh, someone’s just doing this. I’ve just been to England, or Scotland, or America and they were doing this’”* (Policy maker, Participant 33)*.* Disregarding local learning, experience, and needs began to be understood as a distancing and demoralising experience for IAs and frontline staff:

*We’re thinking,* ‘Don’t discount the in-house and the local expertize that we might have, as well as the value in using local examples of innovation and scaling’*, because people relate to that much easier than they would caesareans, [for example], in South America.* (Policy maker, Participant 33).

This reflection led to an attempt to develop a *“ground-up”, “sector-led”* community-of-practice. Policy makers were aware it was important to eschew didacticism and identified the issue of competition between hospitals as *“a really strong theme in terms of what people want to address. I can’t tell you how many times we had doctors, particularly, going, ‘Golly, it was good to find out what our neighbors are doing’*” (Policy maker, Participant 34).

Themed, structured cross-hospital knowledge sharing to enable a more coordinated, wider spread of process improvement ideas became a focus, and emphasis was placed on helping IAs to better integrate process improvement knowledge into hospitals’ operational processes. This was achieved through organizational development tools that tested the depth to which an improvement “mentality” extended into the organization, the governance structures supporting process improvement within the hospital, and the nature and level of organizational learning that was occurring (e.g. triple loop learning). The emphasis was less on an imposed fidelity to process improvement methodologies for the sake of it, and more on distributed learning throughout the organization. While, ultimately, the success of this changed approach is yet to be established, these changes were received enthusiastically by IAs. IAs reported growing in confidence, moving away from a *“fundamentalist”, “quite strict”* approach to improvement (Executive Sponsor, Participant 6) that alienates frontline professionals, and learning how to *“soften”* process improvement methodologies and render them *“more adaptable”* (IA, Participant 20) to the sensibilities of frontline professionals. By this what was meant was a more relaxed approach to using process improvement methodologies. If clinicians emerged from a meeting with a better understanding that the root cause of disappearing bladder scanners was not due to porters failing to return them from another ward, IAs considered this genuinely useful. It opened up the possibility for later conversations about the genuine root cause of the issue, and, more crucially, it exposed clinicians to the Five Whys tool and provided a platform for using it with more fidelity at a later point.

## Discussion

Knowledge brokering is promoted in much healthcare management and implementation science literature as a mechanism that supports service improvement, but we suggest its success is largely shaped by context. A challenge for policy makers, then, is understanding how to foster conditions at the local level that are favourable to knowledge brokers. Our findings suggest this presents policy makers with a conundrum. If knowledge brokers are essentially the creation of policy makers and the perceived conduits of their agenda, their transplantation into health services is by default engineered hierarchically, rather than occurring organically. In these circumstances, knowledge brokers and the ideas they purvey are, fundamentally, an imposition on frontline practice; they do not arise through the perceptions, needs, ingenuity, exigencies, and everyday experiences of medical professionals at the front line.

It has been hypothesized that new ideologies and discourses – new belief systems, that is – tend to have power during exceptionally “unsettled” periods [34] where things are at stake that threaten people’s everyday way of life. Perhaps this accounts for why policy makers’ and executive sponsors’ *“burning platform”* idea has taken hold. If people at the frontline feel the heat, they will be prepared to entertain otherwise objectionable ideas and adapt their practices to suit the context. But our findings show that attempts to manufacture this sense of urgency do not bring intended adopters onside; instead, they tend to foment resentment. And, in any case, people are capable of changing (at least to some degree) the context itself. Under the right conditions – conditions of collaboration, respect, and an authentic felt-need for change – people do not need their context to be changed *for* them [31]. Moreover, other innovation diffusion scholars (e.g. [27]) suggest that meaningful diffusion actually occurs in a direction quite different than a top-down causal flow. As these theorists have observed, [27] if we consider some of the changes that have taken place internationally and become accepted, generally, as the norm on a grand scale (for example, the strengthening of women's rights), little innovation diffusion of any consequence has occurred via powerful actors in charge of formal organisations who attempt to design behavioural settings. Pulling on purse strings and establishing formal rules to shape subordinates’ behaviour probably has some effect, but these kinds of activities have relatively little impact on waves of meaningful change happening across the globe [27]. The most meaningful changes appear to be those that affect people’s everyday lives and occur organically, in seemingly mundane, everyday contexts. Truly influential ideas that alter being and practice “diffuse more as cultural waves than through [orchestrated] point-to-point diffusion” [27]. This is partly what our participant meant when she advocated for *“grassroots”* involvement so that a *“tipping point”* could be created (Policy maker, Participant 24).

But, where are these *“grassroots”* located? In terms of changing healthcare service delivery, the intervention we studied viewed the hospital “shop floor” as the location for seeding change. Change may indeed need to be sown at the practice level within hospital settings, but for medical professionals, grassroots also lie within the occupational and professional bodies that they belong to; these are the local circles in which medical professionals are immersed, as much if not more so than hospitals, to which they do not necessarily have any special allegiance [46]. Policy makers who seek to "arrange” clinicians’ adoption of non-medical ideas via formal organisations such as hospitals are holding imagined levers. They are reaching into bodies that are relatively peripheral to their intended adopters, in terms of their professional allegiance and sense of belonging or citizenship.

A further challenge for policy makers concerns the nature of the knowledge they wish to mobilise. The more foreign the knowledge to be brokered, the more these challenges are exacerbated. The parachuting of outside experts into people’s every day, practical realities is not usually warmly embraced. Policy makers’ distal searches for new ideas, for example in other countries or industry contexts, without regard to the local context, are not typically greeted with enthusiasm. In short, the novelty of distant, abstract solutions is not always beguiling [24]. Novelty can instead underscore the gulfs between the habitual activities of current practice and idealised new practices not borne of the everyday sweat and wisdom of people at the frontline [23]. Being asked to fundamentally rethink the way that things are done in healthcare can repel potential adopters. New knowledge is therefore relatively easy to find, but far more difficult to exploit. As already documented in the literature, the gap between “exploration” and “exploitation” complicates the work of knowledge brokers, and intensifies the need for support at the frontline [8, 47, 48].

With these findings in mind, and drawing upon the innovation diffusion literature discussed earlier, we identify a number of implications for policy makers who seek to integrate new knowledge into public health systems.

First, when it comes to spreading ideas, engineering connections between people simply puts them in contact with each other. This is how diseases are spread, not ideas [23]. As already noted in the literature, “sponsored interaction” between people (e.g. networks or centrally instigated “communities of practice”) can confirm people’s differences as much as their similarities, and can produce conflict, as well as cohesion [23]. When differences are confirmed between “prior and potential adopters”, so too might be the case for disregarding a proposed innovation. Hence, relational interaction can erect boundaries, as well as overcome them; some of the exchanges we observed between knowledge brokers and medical professionals evidence this difficulty. Therefore, relational mechanisms of diffusion, alone, sometimes achieve limited knowledge integration. Yet, communities of practice are seldom thought of in this manner; certainly the participants in our study had not canvassed these potential pitfalls. Existing scholarship suggests that *cultural* linkages need to be fostered, rather than *relational* linkages alone [23]. We concur. This does not mean that the quality of people’s personal relationships and their interpersonal skills do not matter. But cultural linkages ameliorate an over-reliance on the strength of personal relationships and are therefore a protective factor for the sustainability of change. Stark differences between two knowledge domains may or may not pique the interest of potential adopters, but knowledge that resonates with adopters’ underlying belief systems and advances their professional goals is more likely to appeal to a professional community as a whole, and to be integrated into practice. For this reason, seasoned IAs knew to continually bring frontline clinicians’ focus back to the patient; they knew they had to demonstrate how process improvement would improve patients’ experience. They knew they had to appeal to the cultural foundations and *raison d’etre* of the medical profession. IAs and ESs also emphasised the need for policy makers to reach out to professional associations and colleges – those who guard the canons of the medical discipline and determine what counts as legitimate knowledge [23].

Second, it has been noted in the literature that knowledge that is seen by adopters as exotic must be *suitably* explained and “externalised” if it is to be well-received [49]. In our study, likening patients to widgets was heretical; it antagonised healthcare professionals and undermined the brokering of knowledge. Metaphors are not superficial linguistic devices; they carry meaning from one context into another, deliberately or otherwise [50]. Metaphors, therefore, can help or hinder knowledge brokering and must be chosen wisely.

Third, proficiency with the specialised techniques of the body of knowledge that is to be mobilised (*“tooling up”,* as one IA expressed it) is only one important area of mastery. A need for new knowledge begets a need for that knowledge to be absorbed. Equally crucial, therefore, are the interactional, externalization, and cultural skills of those brokering knowledge to the frontline. At the same time, little thought tends to be given to the knowledge integration capabilities of potential adopters – would-be “seekers” of process improvement solutions, in our case medical professionals at the frontline of service delivery. What adopters might need to experiment and change ought not be overlooked. A single-minded focus on skilling up designated “solvers” of problems (e.g. Improvement Advisors) is in vain in the absence of adopters’ capabilities to integrate novel knowledge [47].

Fourth, the most powerful shift in the Program occurred during Phase 4 when policy makers began a more concerted effort to explore *local* (not distant) novel improvements, and demonstrated their appreciation of these improvements. This involved establishing mechanisms (e.g. system-wide show-case events, communities of practice) to “pull up”, rather than “push down”, local examples of excellence, and lift the performance of struggling healthcare organizations in the process [51]. This approach recognized that innovation is already occurring and demonstrated an emergent understanding that valuable knowledge can be diffused laterally, rather than horizontally. Because policy interventions are often instigated and managed from the centre of institutional arrangements, they tend to push knowledge downward. At the local level, change feels imposed, centrally managed, hierarchical, deficits-informed, and marginal to immediate local interests. Downward knowledge integration also communicates to target populations the primacy of ‘structure’, and the subordinacy of individual or local agency. This chafes at the local level and feels intuitively false. It demoralizes target populations and disrespects local ingenuity and inspiration. When policy makers develop mechanisms that enhance the discoverability of local ideas, and support local leaders to externalize and circulate their ideas, local ideas and ingenuity are validated and celebrated, and their impact expanded. Upward knowledge integration changes, rather than submits to, broader forces. Hence, policy makers might do well to reconsider the causal direction of their assumptions as to how change occurs.

Finally, knowledge integration journeys can be long, difficult, and riddled with setbacks. We suggest policy makers would be wise to avoid, where possible, coercive measures to progress the integration of new knowledge. Arduous journeys require resilience, hope, understanding, and support for knowledge brokers and frontline professionals, alike, which policy makers are well-placed to provide [51]. Laggardly behaviour in integrating knowledge is often a sign of difficulty, not obstinacy, (*"a difficult person is a person in difficulty", *is how one of our participants expressed this idea) and benefits from support rather than discipline. If health services’ performance is variable, collaborative mechanisms and incentives are required to redistribute ideas and insights, which extend beyond top down regulation and coercion [52]. Policy makers create conditions that are receptive to knowledge brokering by being friend, not foe, to those on the front line.

To close our discussion, we offer some reflections on the limitations of our study, and how these might inform future research. Some limitations concern the design of the study, which was partially shaped by pragmatics, and others relate to the circumstances we encountered upon entering the field, including limited access to medical professionals, who were the targeted adopters of process improvement.

In terms of our research design, case studies involve “trade-offs” [36]. They do not aim to be statistically representative [35, 36, 44], and therefore the conclusions we draw from our study are conditional. Our case study took place in a Western, liberal democratic, largely urbanised jurisdiction which boasts a high performing, well-funded public health system that is guided by a set of values that may not be shared by other societies [53, 54]. While in our study jurisdiction there is a commitment at the national and state levels to mobilising and brokering knowledge throughout the sector, the jurisdiction's efforts in this regard are relatively new. The situation differs in countries such as the UK, where Academic Health Science Centres are well established and terminology such as “knowledge brokering” has become part of the practitioner lexicon. Moreover, the degree of fit between the “ideologies” of adopters and brokers, in terms of the kinds of knowledge they value, matters enormously [55, 56] and will also vary from context to context. In our particular context, these ideologies tended to be quite incongruent; encouraging the adoption of process improvement knowledge therefore required considerable legitimating work on behalf of the broker. The jurisdiction where our case study was based is therefore embedded within a specific socio-political context, and has a particular historical and experiential knowledge brokering profile. The actors in our study faced knowledge brokering enablers and constraints that might be different in other jurisdictions. In sum, the knowledge-brokering readiness of the context matters, and will differ across jurisdictions. The applicability of our findings to other contexts will therefore vary.

A further design limitation relates to the processual nature of our study and our efforts to track where possible policy makers’ mediation of context over time. It is difficult to educe temporal phases from empirical data, because events are often entwined rather than discrete, and empirical life is dynamic and fluid; to capture empirical reality with richness across time involves “drawing in” to one’s data pool “phenomena that such as changing relationships, thoughts, feelings, and interpretations” [42]. These phenomena are not easy to neatly bundle up and present [42]. Further, people are reflective beings; their own interpretation of their lived experience will change over time [36]. Analysing such phenomena requires a great deal of interpretive and sense-making work. This can be made transparent, but it can never be perfect.

On the matter of our aim to usefully inform policy making, two points warrant discussion. First, because our study was partly historical and partly in real time, and because we did not conduct action research, a further limitation of our design is that we had no “leverage variables” with which to work [36]. By this we mean we were not able to introduce policy strategies that might have acted as a kind of experiment in terms of identifying activities that impact local context. The upshot of this is that there may be critical variables, or “missing components”, that we have not accounted for here [36]. Future studies, with action research designs, might fill this gap. Moreover, in terms of the practical value of our study, we produce broad principles for policy makers, rather than locally-specific advice appropriate for this particular case only. We lose richness and specificity in order to speak to wider audiences. Thus, our research does not yield detailed recommendations like a program evaluation might, and it does not yield for audiences an actual ready-made strategy for implementation. We offer broad principles and pitfalls for policy makers; it is up to policy makers to decide, given the extant context in which they are embedded, whether it will be valuable to transform a given principle into concrete practical actions [36].

In terms of the different perspectives we were able to include in our study, it was our intention to interview frontline medical professionals to gain their personal accounts as intended and actual adopters of process improvement knowledge. However, it proved difficult to recruit medical professionals because of their pressing professional priorities. This is a significant limitation, as we were unable to gain practicing medical professionals’ personal reflections on their experiences of knowledge brokering activities. The majority of our Executive Sponsor participants, however, and even one of our Improvement Advisors, were also medical professionals, albeit ESs acted in (usually) purely senior management roles and were removed from the front line. While their views are not equivalent to their practicing colleagues, their reflections on knowledge brokering and adopters’ responses were nevertheless infused with an understanding of the “clinical mentality” [57] of medical professionals and the pressures they face, and they were very much aware of the importance of professional jurisdiction [58], and the complexities this created for knowledge brokering. Importantly, we were able to offset this limitation with the considerable amount of observational field work we conducted. In some cases, we were able to observe knowledge brokering taking place, and adopters’ actual responses to these efforts in situ and in real time. In short, we were able to see where “the rubber of theory hits the road of reality”, at the “coalface” [29] where knowledge brokering takes place. These were informative data collection opportunities, given what people say and what people do, can be two different things. This also meant, incidentally, that we were able to “corroborate” (or otherwise) the accounts of our knowledge broker participants.

Finally, and in our view most consequentially, in terms of future research, our study is underpinned by a theoretical assumption that warrants challenging in the future. Useful though our theoretical lens may be, we have conceptually positioned policy makers as mediators of knowledge brokers’ local contexts. This contains an assumption that context is shaped through “top down” processes. But our study suggests that it may be fruitful to challenge this assumption and start with faith in the capability of those at the frontline to adapt their contexts themselves. Indeed, those “on the ground” might be perfectly placed to shape the way that their peers and decision-makers “above” them think, if the right supports, stimuli, and mechanisms are put in place to facilitate the lateral and upwards flow of ideas.

## Conclusion

We have studied the role that local context plays in knowledge brokering, and policy makers’ efforts to shape this context so that it is more conducive to the diffusion and adoption of good ideas. Our case study traces the movement of an intervention conceived outside of the local context and at first mobilized via top-down governance and policy processes. The journey taken was one of adaptation, with policy makers giving more explicit consideration to the local context where knowledge brokering takes place, as time wore on. A major reflection of policy makers involved recognizing the ineffectualness of purely top-down diffusion processes, which sometimes created a fractious context for knowledge brokers and frontline medical professionals. Emerging from our study is the need to reconsider policy makers as the sole “mediators” of the local knowledge brokering context. Policy makers play an important role in this regard, but not the only role. Rather, consideration needs to be given to the mechanisms and supports that foster lateral and bottom-up change, which is why we urge policy makers to “pull knowledge up”. If embodied, contextualized, practical know-how were never sought, attended to, and “amplified” [59], there would be no good ideas to broker in neighbouring or distal contexts. Implications such as these suggest it might be useful for policy makers to slightly reposition the role of knowledge brokers when designing interventions. Perhaps knowledge brokers might be usefully conceived as local scouts and aids “on the ground”, with a role in facilitating lateral and upwards knowledge flow, as well as facilitating the more traditional “downward” direction of brokering. Context is created by causal currents that run in many directions, not one; all are worthy of exploration. 

## Data Availability

The detailed nature of the qualitative research means participating individuals and organisations cannot be anonymized in the primary data set. Raw data are therefore not publicly available to preserve individuals’ privacy as per the conditions of our ethical approval and our undertaking with our participants during the recruitment process. The chief investigators of the project will grant access to interim and final project reports upon reasonable request.

## Abbreviations

IA: Improvement Advisor
ES: Executive Sponsor
